# Minimising Young Children’s Anxiety through Schools (MY-CATS): statistical analysis plan for a cluster randomised controlled trial to evaluate the effectiveness and cost-effectiveness of an online parent-led intervention compared with usual school practice for young children identified as at risk for anxiety disorders

**DOI:** 10.1186/s13063-022-06899-1

**Published:** 2022-12-28

**Authors:** Benjamin G. Jones, Tessa Reardon, Cathy Creswell, Helen F. Dodd, Claire Hill, Bec Jasper, Peter J. Lawrence, Fran Morgan, Ronald M. Rapee, Mara Violato, Anna Placzek, Obioha C. Ukoumunne

**Affiliations:** 1 NIHR ARC South West Peninsula (PenARC), University of Exeter, Exeter, UK; 2 Exeter Clinical Trials Unit (ExeCTU), University of Exeter, Exeter, UK; 3 Departments of Experimental Psychology and Psychiatry, University of Oxford, Oxford, UK; 4 College of Medicine and Health, University of Exeter, Exeter, UK; 5 School of Psychology & Clinical Language Sciences, University of Reading, Reading, UK; 6 Parents and Carers Together, Suffolk, UK; 7 Centre for Innovation in Mental Health, School of Psychology, University of Southampton, Southampton, UK; 8 Square Peg, East Sussex, UK; 9 Centre for Emotional Health, School of Psychological Sciences, Macquarie University, Sydney, Australia; 10 Health Economics Research Centre, Nuffield Department of Population Health, University of Oxford, Oxford, UK

**Keywords:** Statistical analysis plan, Cluster randomised trial, School-based, Anxiety, Clustering, Random effects, Mediation

## Abstract

**Background:**

The Minimising Young Children’s Anxiety through Schools (MY-CATS) trial is being conducted to determine whether an online evidence-based parent-guided cognitive behavioural therapy intervention in addition to usual school practice is effective and cost-effective compared with usual school practice in reducing anxiety disorders in children aged 4–7 deemed ‘at risk’ of anxiety disorders. This update article describes the detailed statistical analysis plan for the MY-CATS trial and reports a review of the underpinning sample size assumptions.

**Methods and design:**

The MY-CATS study is a two-arm, definitive superiority pragmatic parallel group cluster randomised controlled trial in which schools will be randomised 1:1 to receive either the intervention (in addition to usual school practice) or the usual school practice only. This update to the (published) protocol provides a detailed description of the study methods, the statistical principles, the trial population and the planned statistical analyses, including additional analyses comprising instrumental variable regression and mediation analysis.

**Trial registration:**

ISRCTN82398107. Prospectively registered on 14 January 2021

## Introduction

### Background and rationale

This article describes the detailed statistical analysis plan for the Minimising Young Children’s Anxiety through Schools (MY-CATS) trial and has been produced in accordance with published guidance on the content of statistical analysis plans [12]. This article does not contain detail of the planned health economic evaluation.

Anxiety disorders are the most common mental health disorders experienced across the lifespan (Kessler et al., [23]). Half of all lifetime cases begin by age 11 (Kessler et al., [23]), and the estimated prevalence amongst children and adolescents worldwide is 6.5% (Polanczyk, Salum, Sugaya, Caye, & Rohde, [31]). Children with anxiety disorders are more likely than their peers to experience ongoing anxiety problems and other serious mental health disorders and have reduced educational and employment opportunities (e.g. Copeland, Angold, Shanahan, & Costello, [8]). Due to the high prevalence, anxiety disorders have higher societal costs than any other mental health disorder (Fineberg et al., [9]). Intervening before anxiety disorders develop would minimise the consequences for children, their families and society.

There is evidence that universal approaches that target a whole population may not benefit children with high anxiety symptoms, who most need the intervention (Stallard et al., [41]). An alternative approach is to target interventions at children who are *most* likely to develop anxiety disorders; emerging evidence supports the value of this approach (Lawrence, Rooke, & Creswell, [25]).

The MY-CATS trial evaluates the effectiveness and cost-effectiveness of an online evidence-based parent-guided cognitive behavioural therapy intervention called OSI (Online Support for Intervention for child anxiety) with therapist support in addition to usual school practice (from hereon ‘OSI’ means ‘OSI + usual school practice’) compared with usual school practice in reducing anxiety disorders in children aged 4–7 deemed ‘at risk’ of anxiety disorders. Further details of the background to the study are available in the protocol [34].

### Objectives

The goals of the study are:

1. To evaluate the effectiveness and cost-effectiveness, relative to usual school practice, of the provision of OSI for children (aged 4–7) identified as at risk for anxiety disorders, on the basis of screening positive for one or more risks
2. To identify the characteristics of children who do and do not benefit from the intervention and the mechanisms of change from the intervention

The primary objective of the study is to compare the presence of an anxiety disorder at 12 months post-randomisation (the primary outcome) amongst children who screen positive on one or more risk factors between those in schools allocated to the intervention arm and those in schools allocated to usual school practice.

Secondary objectives are to (i) compare anxiety symptoms, related interference and externalising symptoms and additional intervention targets at 12 weeks and 12 months post-randomisation amongst children who screen positive on one or more risk factors in schools allocated to the intervention arm to those in schools allocated to usual school practice; (ii) examine potential moderators and mediators of the intervention effect on the primary outcome; (iii) evaluate experiences of systematic screening and the supported parent-led online intervention and (iv) estimate the cost-effectiveness of the intervention compared to usual school practice, 12 months post-randomisation.

## Study methods

### Trial design

The study is a two-arm, definitive superiority pragmatic parallel group cluster randomised controlled trial in which schools (clusters) will be randomised 1:1 to receive either the intervention or the usual school practice. In recruited schools, parents of all infant school-aged children (aged 4–7) in classes in three year groups (reception, year 1 and year 2) selected through random sampling (2 classes per year group per school) will be invited to complete risk screening questionnaires. Children who screen positive on the basis of child anxiety symptoms, behavioural inhibition or parent anxiety symptoms (or any combination of the three) will be eligible for the trial. A maximum of one child per family/household will be eligible for the trial, and where more than one child in a family/household screens positive on at least one risk factor, one child will be invited to take part on the basis of screening scores. Screening, participant recruitment and baseline assessment will take place prior to randomisation. Following this, schools will be randomised to either the intervention or the usual school practice arm. Parents of children in schools allocated to the intervention arm will receive the intervention, an online parent-guided cognitive behaviour therapy intervention for child anxiety, supported by brief telephone sessions with a Children’s Wellbeing Practitioner. The intervention comprises 7 weekly sessions starting as soon as possible after randomisation, followed by a review session 4 weeks later. Schools in both arms will continue to provide any usual support to children and parents throughout. Data collection occurs at screening and baseline (pre-randomisation), as well as 6 weeks, 12 weeks and 12 months post-randomisation.

### Class sampling, randomisation and blinding

In recruited schools with more than two classes in any of the three eligible year groups, a computer-based random sampling procedure is being used to select exactly two classes from each year group whose pupils will be invited to take part in eligibility screening. The programme used for sampling classes was written in R software [33] by the trial statistician.

Batch 1 was recruited and randomised between February 2021 and May 2021, and each subsequent batch is being recruited and randomised in a new school term. Allocation is stratified by the level of deprivation, determined according to whether the proportion of children eligible for free school meals in the school falls above or below the national median of 15.8% for primary schools in 2019 [44].

The programme for generating the 1:1 randomisation sequence was written by the trial statistician using R software. Specifically, a blocked randomisation list using block sizes of two and four was created for each deprivation category. In order to minimise any imbalance between trial arms in terms of the number of pupils, the schools in each batch are ordered by the number of recruited pupils before being allocated a trial arm according to the allocation lists. This approach has been designed to balance on the key prognostic factor of deprivation, whilst minimising the potential for imbalance in the number of pupils allocated to each trial arm.

An independent statistician otherwise unassociated with the project is undertaking both the random sampling of classes and the randomisation of schools.

It will not be possible to maintain trial statistician blinding during statistical analysis because much of the analysis relies on unblinded data, for example auxiliary variables based on levels of intervention engagement used in multiple imputation. However, every attempt will be made to maintain blinding of the trial statistician throughout trial delivery.

### Sample size

Our original target sample size was 1080 ‘at risk’ children from 60 schools (30 schools (clusters) and 540 children in each trial arm). To achieve this, we planned to invite six classes per school (two classes in each of three year groups) to take part in screening (estimated 30 children per class, 10,800 children in total). Where schools have more than two classes in each year willing to take part, classes will be chosen randomly, using computer-generated random numbers. The target size is large enough to detect a reduction in the presence of anxiety disorders at 12 months (primary outcome) from 50% (control arm) to 35% (intervention arm) with 90% power at the 5% (2-sided) level. This difference would be meaningful to detect and is in line with outcomes reported in previous child anxiety prevention trials with positive findings (e.g. [28]). This sample size calculation assumes (i) 50% of invited children participate in screening (5400 of 10,800), (ii) 20% of those who participate in screening screen positive, and (iii) 80% of those that screen positive complete the 12-month follow-up. It also allows for clustering within schools, assuming an intra-cluster (intra-school) correlation coefficient of 0.05, and assumes a fixed cluster size. Fifty percent participation is a conservative estimate based on a recent UK school-based study in which 72% of parents responded to similar class-wide screening questionnaires [10]. Previous studies indicate 10–15% will score above the cut-off on each single screen (Bayer et al., [2]; Löwe et al., [26]; [39]); we estimate 20% will screen positive on at least one risk factor (from Hudson et al., [20]). The median intra-cluster (intra-school) correlation coefficient in a recent systematic review of school-based cluster randomised trials was 0.028 (Parker et al, [30]). Our assumed value of 0.05, therefore, leans on the side of caution.

As schools and participants are recruited in four separate cohorts, we have the opportunity to monitor recruitment rates and review some of the sample size assumptions (screening participation rate, screen-positive rate and cluster sizes, including variability in cluster size) after each cohort. If there are insufficient participants recruited in initial cohorts, it is possible to increase the total number of schools as required. If we need to increase the number of schools, the total number of trial participants we will require to detect the same effect size may also change.

#### Review of sample size assumptions following initial recruitment

Across the first two cohorts of schools, we recruited 35 schools altogether. Participating schools had an average of 27 pupils per participating class. In these 35 schools, 983 children were screened (17% of invited children), and 360 children screened positive and enrolled on the trial (37% of children who participated in screening), with a mean (SD) cluster size of 10.3 (7.0), giving an estimated coefficient of variation in cluster size of 0.68. If we continue to recruit at the current rate, we expect to recruit approximately 611 trial participants in total from 60 schools. This assumes 9720 children will be invited to take part in screening (27 per class), 17% (*n*=1652) will take part in screening, 37% (*n*=611) of those screened will screen positive and enrol on the trial, and 80% (*n*=489) of those who enrol on the trial will complete the 12-month follow-up. Assuming an intra-cluster correlation of 0.05 and a coefficient of variation in cluster size of 0.68, this would only provide 74% power to detect a reduction in the presence of anxiety disorders at 12 months from 50% (control arm) to 35% (intervention arm).

To ensure we are able to detect the same effect size with at least 85% power at the 5% (2-sided) level, we will increase the total number of schools above the original target, based on the following amendments to our initial assumptions:

- A reduction of the assumed percentage of children amongst those invited to screening who take part from 50 to 17%
- An increase in the assumed percentage of children amongst those who took part in screening who screened positive and enrol on the trial from 20 to 37%
- A variable cluster size, assuming a coefficient of variation in cluster size of 0.68

Based on the current recruitment rate, we are aiming to include 86 schools in total, which would allow us to recruit 876 trial participants, and provide approximately 88% power at the 5% (2-sided) level to detect a reduction in the presence of anxiety disorders at 12 months from 50 to 35%. This assumes 13,932 children will be invited to take part in screening (27 per class), 17% (*n*= 2368) will take part in screening, 37% (*n*= 876) of those screened will enrol on the trial, and 80% (*n*=701) of those who enrol on the trial will complete the 12-month follow-up.

### Framework

The study is a superiority trial and the hypothesis testing framework is specified in such a way that a definitive conclusion may be obtained with regard to the primary trial objective. Specifically, the null hypothesis is that there is no difference in the proportion of children with an anxiety disorder between the intervention arm and the usual school practice arm at 12 months post-randomisation. Whilst hypothesis testing will also be undertaken to address some of the secondary objectives, the results of these will be interpreted as exploratory only and may be used to inform future research or hypothesis generation.

### Statistical interim analyses and stopping guidance

There are no planned statistical interim analyses or stopping criteria as part of this study.

### Timing of final analysis

The final statistical analyses will be undertaken once all participants have provided follow-up data at 12 months (or have been withdrawn from the study or deemed lost to follow-up) and once all data queries have been satisfactorily resolved and the database locked.

### Timing of outcome assessments

Data collection occurs at screening (T-1) in order to determine eligibility, as well as at baseline (pre-randomisation) (T0), 6 weeks (T1), 12 weeks (T2) and 1 year (T3) post-randomisation. Every endeavour will be made to collect outcome data within a pre-specified window of the relevant timepoint (at 6 weeks: +2 weeks; at 12 weeks: +3weeks; at 12 months: +12 weeks). Full details of timings of data collection are provided in Table 1.

**Table 1:** Timing of data collection

**Outcome**	**Subject**	**T-1** **screening**	**T0** **baseline**	**T1** **6 weeks**	**T2** **12 weeks**	**T3** **1 year**
Child anxiety disorder diagnosis status	Child					X
PAS (child anxiety symptoms)	Child	X	X	X	X	X
Approach subscale—STSC (behavioural inhibition)	Child	X	X	X	X	X
GAD-7 (parent anxiety symptoms)	Parent	X	X	X	X	X
CALIS-PV (impact of child anxiety)	Child		X		X	X
SDQ-E (externalising symptoms)	Child		X		X	X
Parent motivation	Parent		X			
POS (parent overprotection)	Parent		X	X	X	X
PSCS-self-efficacy subscale (parenting self-efficacy)	Parent		X	X	X	X
RULES (child tolerance of uncertainty)	Child		X	X	X	X
CQ-P (child coping efficacy)	Child		X	X	X	X
CAMP (child behavioural avoidance)	Child		X	X	X	X
CHU-9D (child health-related quality of life )	Child		X		X	X
EQ-5D-5L (parent health-related quality of life)	Parent		X		X	X
CSRI (individual resource use)	Parent and child		X		X	X
Time logs (resources used in screening and the intervention)		X (onwards)				
Adverse experiences	Parent and child		X		X	X
Acceptability	Parent and child		X		X	X

## Statistical principles

### Confidence intervals and *p*-values

All hypothesis testing will be undertaken at the 5% (two-sided) statistical significance level. The estimates of all between-group comparisons will be presented with their corresponding 95% confidence intervals (CIs) and *p*-values. For the binary primary outcome of the presence/absence of child anxiety disorder diagnosis, the intervention effect will be quantified using the odds ratio. For continuous secondary outcomes, mean differences will be reported alongside standardised mean differences. As the study includes only a single primary outcome, no adjustment will be made for multiple testing, and the secondary outcomes will be treated only as exploratory and interpreted in the context of hypothesis generation for future research.

### Adherence and protocol deviations

The intervention consists of seven online modules for parents, supported by brief telephone sessions with a Children’s Wellbeing Practitioner (CWP), and a follow-up review session 4 weeks later. The 6-week (T1) data collection timepoint will take place part way through the intervention, and the 12-week (T2) data collection timepoint will take place after the expected completion of the intervention. CWPs will follow highly structured and standardised guidance on how to support parents and will receive weekly supervision from a clinical psychologist. Adherence will be closely monitored by the supervising clinical psychologists, facilitated by the use of audio recordings of telephone sessions in supervision.

Usage of the online modules will be captured through the intervention platform (including completion of modules and optional interactive activities), and CWPs will record telephone support session attendance. Intervention adherence will be summarised appropriately, including mode of access (pc/laptop, phone, tablet), login times (night, day, evening), non-optional pages viewed, page views per module, percentage of in-module questions and quiz questions answered, and the percentage of quiz questions answered correctly.

For the purposes of a Complier Average Causal Effect (CACE) secondary analysis (see the ‘Additional analyses’ section), a participant will be classified as a complier if they complete at least the first five online modules (modules 0–4).

### Analysis populations

The primary analyses of the primary outcome and all secondary outcomes will be undertaken in line with the principles of intention-to-treat (ITT), where participants will be analysed according to the trial arm that their school was randomly assigned to, regardless of the intervention actually received. These analyses will be undertaken on the full ITT population (i.e. by imputing any missing data), with further details of the planned multiple imputation strategy outlined below. A complete case analysis of the ITT population (i.e. analysis of the observed data only) will also be undertaken for the primary outcome and each of the secondary outcomes for the purposes of sensitivity analyses.

## Trial population

### Screening data

After school-level recruitment and sampling of classes to be invited to participate, children will be deemed eligible to participate in the study if they screen positive on at least one of (i) child anxiety symptoms, (ii) behavioural inhibition or (iii) parental anxiety symptoms. Amongst those children eligible for the trial, we will report summary statistics of each of the measures used to determine this eligibility and report this separately for children who subsequently agree to participate in the study and those who do not agree to participate (including those who decline and those who do not respond) to assess whether or not there are any noteworthy differences between participants and eligible non-participants.

### Eligibility

Schools are eligible to take part in the study if they are mainstream primary/infant schools in England with a minimum of two classes per target year group. All children within the randomly sampled classes within reception, year 1 and year 2 are eligible to participate in screening, with one parent/carer completing the screening questionnaires for each child. Children are eligible to participate in the trial if they screen positive on at least one of the three criteria outlined above. A maximum of one child per family/household is eligible to take part in the trial; where more than one child in a family/household screens positive on at least one risk, the child with the highest Preschool Anxiety Scale (PAS) score (child anxiety symptoms) is eligible. If two or more children in the family/household have equal PAS scores, the child with the highest STSC-Approach subscale score is eligible.

Children will be deemed ineligible to participate in the trial if their parent or carer does not have sufficient use of English to provide consent, complete measures and/or take part in the intervention or if they do not have frequent access to the Internet, either at home or elsewhere.

### Recruitment

Recruitment to the trial will be reported via a CONSORT flow diagram. Specifically, the number of schools approached, eligible and consented, and the number of participants invited to take part in screening, screened and eligible for the trial will be reported. In addition, the number of schools and the number of trial participants recruited and randomised to each trial arm and assessed at each of the data collection timepoints (screening, baseline, 6 weeks, 12 weeks and 1 year) will be reported. The number of participants and/or schools withdrawn or lost to follow-up between each timepoint will also be reported, alongside reasons where possible. The mean and standard deviation of the cluster size will also be reported, by allocated group, at each timepoint, in line with the recommendations within the CONSORT extension to cluster randomised controlled trials [5].

### Withdrawal/follow-up

Every attempt will be made to minimise withdrawal and loss to follow-up by following procedures used in a recent UK school-based trial [10].

Both individual- and cluster-level loss to follow-up will be reported at each data collection timepoint, by allocated group and overall. A participant will be deemed lost to follow-up at a given timepoint if the study team are unable to facilitate data collection within the pre-specified time frame of the data collection timepoint (at 6 weeks: + 2 weeks; at 12 weeks: +3 weeks; at 12 months: + 12 weeks), but this will not preclude the participant providing data at later timepoints, if applicable.

### Baseline characteristics

Child-, parent- and school-level baseline (pre-randomisation) characteristics will be presented by allocated group, and overall. Continuous characteristics will be summarised using means and standard deviations (or medians and interquartile ranges), and categorical characteristics will be summarised using counts and percentages. Each of the secondary outcomes, as well as the results of the screening measures (child anxiety symptoms, behavioural inhibition and parental anxiety), will also be summarised.

## Analysis

### Outcome definitions—primary outcome

The primary outcome is the presence (yes/no) of an anxiety disorder at 12 months post-randomisation. At the 12-month assessment, psychology graduates (blind to trial arm status and trained to a high level of inter-rater reliability) will assess for child anxiety disorders using the Anxiety Disorder Schedule—Child Version—Parent Interview (ADIS-P) [38] over the telephone or via video call [27].

### Outcome definitions—secondary outcomes

#### Child anxiety symptoms

Assessed using the PAS [39], a 28-item, parent-report scale, where scores can range from 0 to 112 (each item scored from 0 to 4), with higher scores indicating a higher overall level of anxiety.

#### Behavioural inhibition

Assessed using the approach subscale of the Short Temperament Scale for Children (STSC) [ 32, 37], a 7-item, parent-report scale, where total scores can range from 7 to 42 (each item scored from 1 to 6), with higher scores representing a greater degree of behavioural inhibition.

#### Parental anxiety

Assessed using the Generalised Anxiety Disorder Scale (GAD-7) [40], a 7-item questionnaire with a total score that can range from 0 to 21 (each item scored from 0 to 3), where higher scores represent a greater degree of anxiety.

#### Parental overprotection

Assessed using the Parental Overprotection Scale (POS) [7], a 19-item parent-report outcome with a total score that can range from 0 to 76 (each item scored from 0 to 4) with higher scores representing a higher degree of parental overprotection.

#### Parenting self-efficacy

Assessed using the self-efficacy subscale of the Parenting Sense of Competence Scale (PSCS) [21], a 7-item parent-report measure with a total score ranging from 7 to 42 (each item scored from 1 to 6), with higher scores indicating a greater degree of parenting self-efficacy.

#### Child coping efficacy

Assessed using an adapted version of the Coping Questionnaire (CQ-P) [22]. Parents rate their child’s coping in up to three anxiety-provoking situations (each item scored from 1 to 7). Coping scores for the first item (scores ranging 1 to 7) will be used in analyses, with higher scores representing a higher degree of child coping efficacy.

#### Child behavioural avoidance

Assessed using the Child Avoidance Measure—Parent Report (CAMP) [46], an 8-item questionnaire with a total score ranging from 0 to 24 (each item scored from 0 to 3), with higher scores indicating a greater degree of child behavioural avoidance.

#### Child intolerance of uncertainty

Assessed using the Responses to Uncertainty and Low Environmental Structure (RULES) [36], a parent-report, 17-item scale with a total score ranging from 17 to 85 (each item scored from 1 to 5), with higher scores indicating a greater intolerance of uncertainty.

#### Child anxiety-related interference

Assessed using the Child Anxiety Life Interference Scale—Preschool version (CALIS-PV) [14], an 18-item parent-report measure with scores ranging from 0 to 72 (each item scored from 0 to 4), with higher scores indicating a higher degree of anxiety-related interference.

#### Child externalising symptoms

Assessed using the externalising score from the Strengths and Difficulties Questionnaire (SDQ) [15], calculated as the total of the conduct and hyperactivity subscales comprised of 10 items each scored from 0 to 2. The total score ranges from 0 to 20, with higher scores indicating greater externalising symptoms.

#### Health-related quality of life

Child health-related quality of life was assessed using the Child Health Utility 9D (CHU-9D) [43], and parent health-related quality of life was assessed using the EQ-5D-5L [19] and used to estimate quality adjusted life years. This outcome will be analysed as part of the health economic analysis, along with the resource use data collected through an updated version of the Client Service Receipt Inventory (CSRI) [3].

#### Acceptability of trial participation

Assessed at baseline, 12 weeks and 12 months using a bespoke parent or carer questionnaire, including questions about any adverse experiences relating to participation in the trial.

### Pre-specified moderators

The following baseline moderators of the primary outcome will also be explored:

- *Child year group (reception versus year 1 versus year 2)*
- *Child gender (female versus male versus others)*
- *Child ethnicity (white British versus others)*
- *Parent gender (female versus male versus others)*
- *Parent ethnicity (white British versus others)*
- *Family socioeconomic status (bottom quintile of the index of multiple deprivation rank versus remaining four quintiles)*
- *Child anxiety symptoms*: assessed at screening using the PAS and dichotomised according to the screen-positive criteria
- *Behavioural inhibition:* assessed at screening using the STSC-approach subscale and dichotomised according to the screen-positive criteria
- *Parental anxiety*: assessed at screening using the GAD-7 and dichotomised according to the screen-positive criteria
- *Total number of risk factors:* between 1 and 3 (child anxiety symptoms, BI and parental anxiety), coded as categorical
- *Combination of risk factors:* Further exploration of risk factor combinations will be undertaken if there is an indication that they are potential moderators of the primary outcome
- *Parent motivation*: assessed using a bespoke parent-report questionnaire comprising six items each scored 1 to 5, with higher scores representing a higher level of motivation. Motivation will be categorised as ambivalent (scores 6–18), partially motivated (scores 19–23) or motivated (scores 24–30).

### Pre-specified mediators

The risk factors (child anxiety symptoms, behavioural inhibition and parental anxiety) will be explored as possible mediators of the primary outcome. In addition, further intervention targets will be explored as possible mediators of the primary outcome. Specifically, parental overprotection (using the Parental Overprotection Scale (POS)), parenting self-efficacy (using the Parent Sense of Competence Scale (PSCS)), child behavioural avoidance (using the Child Avoidance Measure—Parent Report (CAMP)), child coping efficacy (using the Child Coping Questionnaire—Parent Report (CQ-P)) and child intolerance of uncertainty (using the Responses to Uncertainty and Low Environmental Structure (RULES)) will all be assessed for their possible role as mediator of the relationship between the intervention and the primary outcome. All potential mediators will be examined at 12 weeks; initially, the intention was to also explore mediators at 6 weeks, but subsequently, the decision was taken to present 6-week data for descriptive purposes only.

### Blinding status

As the primary outcome will be collected via interview, data on whether or not the assessors/supervisors of this outcome inadvertently became unblinded during the interview will be collected. Specifically, for each interview, the assessor will be asked whether or not they became aware of the allocated group of the interviewee.

### Analysis methods

The primary analysis of all outcomes will be undertaken in line with the principle of intention-to-treat with participants analysed according to the trial arm their school was randomised to. Marginal models using generalised estimating equations with robust estimates of the standard error (specifying an exchangeable correlation structure) will be used to analyse the primary outcome, and mixed effects linear regression models, with random effects at the school (cluster) level, will be used to analyse the continuous secondary outcomes. These methods allow for the correlation between responses from the same cluster. The intervention effect will be presented as an odds ratio for binary outcomes, and as a mean difference for continuous outcomes.

Both unadjusted (crude) and adjusted estimates of the intervention effect will be presented, with the latter adjusted for school-level free school meal status (the stratification factor) and cluster size as a continuous variable to reflect its role in the randomisation process, as well as for the baseline value of the outcome (for continuous outcomes only), where this is available, gender, year group, cohort and the decile of the index of multiple deprivation rank (as a continuous predictor) as a measure of socioeconomic status. The nature of the relationship between the adjustment factors and the outcome will be examined prior to the main analysis. The main findings will be based on the results of the adjusted models. Visual inspection of baseline characteristics at both the individual and cluster levels will be undertaken, and additional adjustments for any characteristics deemed to be substantially different will be considered for the purposes of sensitivity analyses.

The primary analyses of all outcomes will be of data obtained using multiple imputation (see the ‘Missing data’ section for further details) and combined using Rubin’s rules. Analyses based on complete case data only will also be presented for the purposes of additional sensitivity analyses.

For each outcome at each timepoint, both crude estimates of the intra-cluster (intra-school) correlation coefficients (ICCs) from unadjusted models (as these are the values most useful for researchers planning future studies) and fully adjusted ICCs will be presented.

Tests of interaction effects will be used to identify potential factors that moderate the effect of the intervention on the primary outcome (see the ‘Pre-specified moderators’ section for a list of these factors). Treatment effect estimates, alongside 95% CIs, will be presented for each subgroup. However, any signals of moderating effects will be interpreted as exploratory only and presented for their potential to generate hypotheses for future research. If any additional potential moderators become apparent, they will also be considered for further exploration through tests of interaction, although the results of these analyses will be clearly labelled as ‘post hoc’.

Model assumptions will be visually assessed using appropriate diagnostic plots. In any cases where the assumption of normality appears to be substantially violated, bootstrapping methods will be used in order to ensure the robustness of the results.

### Missing data

For each primary and secondary outcome, at each follow-up timepoint, the amount of missing data will be summarised separately by trial arm status and overall, by reporting the percentage of missing observations.

Where there is partial missing data for outcomes with multiple items, the total score will be ‘scaled up’ based on the average score across the non-missing items. For each outcome, there is a minimum number of items that should be completed in order to allow valid extrapolation of the total score. These procedures will be followed unless the manuals for the relevant measures suggest an alternative approach to the management of missing data. Further detail of the management of missing data is shown in Table 2.

**Table 2:** Outcome scoring instructions and management of missing item-level data

	**Scoring**	**Managing missing items**
**Screening questionnaires**		
Preschool Anxiety Scale (PAS)	• 28 items • Items scored 0–4 0= Not true at all 1=Seldom true 2=Sometimes true 3=Quite often true 4=Very often true • Total score: sum of all items; range 0–112 • Screen positive: total score ≥34	No missing items allowed
Approach subscale of the Short Temperament Scale for Children (STSC-A)	• 7 items • Items scored 1–6 1=Almost never 2=Not often 3=Variable, usually does not 4=Variable, usually does 5=Frequently 6=Almost always • Items 3–6 reverse scored • Total score: sum of all items; range 7–42 • Screen positive: total score ≥30	No missing items allowed
Generalised Anxiety Disorder Scale (GAD-7)	• 7 items • Items scored 0 to 3 0= Not at all 1=Several days 2=More than half the days 3=Nearly every day • Total score: sum of all items; range 0–21 Screen positive: total score ≥8	No missing items allowed
**Questionnaire outcome measures**		
Preschool Anxiety Scale (PAS)	As above	Where ≥ 75% items completed (≥21 items), calculate prorated total score using completed items Where ≥8 missing items, treat measure as missing
Approach subscale of the Short Temperament Scale for Children (STSC-A)	As above	Where 6 items completed, calculate prorated total score using completed items Where ≥2 missing items, treat measure as missing
Generalised Anxiety Disorder Scale (GAD-7)	As above	Where 6 items completed, calculate prorated total score using completed items Where ≥2 missing items, treat measure as missing
Child Anxiety Life Interference Scale—Preschool version (CALIS-PV)	• 18 items • Items scored 0–4 0=Not at all 1=Only a little 2=Sometimes 3=Quite a lot 4=A great deal • Total score: sum of all items; range: 0–72 • Higher scores indicate greater interference	Where ≥ 75% items completed (≥14 items), calculate prorated total score using completed items Where ≥5 missing items, treat measure as missing
Strengths and Difficulties-Externalising Scale (SDQ-E)	• 10 items (items 2, 5, 7, 10, 12, 15, 18, 21, 22, 25) • Items scored 0–2 0=Not true 1=Somewhat true 2=Certainly true • Items 7, 21 and 25 reverse scored • Total score: sum of all items; range: 0–20 • Higher scores indicate a higher level of externalising symptoms	Where ≥3 items completed for each of the two 5-item subscales, calculate prorated subscale score using completed items before calculating the total score Where ≥2 missing items on either subscale, treat measure as missing
Parent Overprotection Scale (POS)	• 19 items • Items scored 0–4 0=Not at all 1=A little 2=Somewhat 3=Quite often 4=Very often • Total score: sum of all items; range: 0–76 • Higher scores indicate greater parental overprotection	Where ≥ 75% items completed (≥15 items), calculate prorated total score using completed items Where ≥5 missing items, treat measure as missing
Parenting Sense of Competence Scale-self-efficacy scale (PSOC-SE)	• 7 items • Items scored 1–6 1=Strongly disagree 2=Disagree 3=Somewhat disagree 4=Somewhat agree 5=Agree 6=Strongly agree • Total score: sum of all items; range: 7–42 • Higher scores indicate greater parenting self-efficacy	Where 6 items completed, calculate prorated total score using completed items Where ≥2 missing items, treat measure as missing
Child Avoidance Measure (CAMP)	• 8 items • Items scored 0–3 0=Never, almost never or not an issue 1=Sometimes 2=Often 3=Almost always • Total score: sum of all items; range: 0–24 • Higher scores indicate greater child avoidance	Where ≥6 items completed, calculate prorated total score using completed items Where ≥3 missing items, treat measure as missing
Responses to Uncertainty and Low Environmental Structure (RULES)	• 17 items • Items scored 1–5 1=Not at all 5=Very much • Total score: sum of all items; range: 17–85 • Higher scores indicate higher greater intolerance of uncertainty	Where ≥ 75% items (≥ 13 items) completed, calculate prorated total score using completed items Where ≥5 missing items, treat measure as missing
Coping Questionnaire (CQ-P)	• Up to 3 items • Items scored 1–7 1= Not at all able to help themself 7 =Completely able to help themself • Score: first item score; range 1–7 • Higher scores indicate a higher level of coping	Where the first item is missing, treat measure as missing
Additional questionnaire measure		
Motivation questionnaire	• 6 items • Items scored 1–5 1=Strongly disagree 2=Disagree 3=Neither agree nor disagree 4=Agree 5=Strongly agree • Item 4 reverse scored • Total score: sum of all items; range 6–30 • Total score 6–18 = ‘ambivalent’ Total score 19–23 = ‘partially motivated’ Total score 24–30 = ‘motivated’	Where at least 5 items are completed, calculate the prorated total score using completed items

The primary analyses of all outcomes will be undertaken on imputed data. These imputed data will be obtained using a joint modelling multiple imputation approach based on a multivariate linear mixed effects model that accounts for clustering in the data by including random effects at the school level. The imputation model will include all outcomes at all timepoints, allocated group, the variables used to balance the randomisation, any additional adjustments and, if possible, the pre-specified moderators and mediators of the primary outcome. We will also aim to include in the imputation model measures of health-related quality of life (i.e. utilities derived from the CHU 9D and EQ-5D-5L, for child and parent, respectively), healthcare and wider societal costs. However, we acknowledge that we may not be able to receive the completely processed health economic data in a timely enough fashion for inclusion in the imputation procedure and may therefore need to modify the planned specification of the imputation model in relation to these variables. The number of completed OSI modules will be used as an auxiliary variable, set to zero for participants in the usual school practice arm. Dummy variables will be used to facilitate the inclusion of categorical variables, and imputation of both binary and categorical variables will follow the rule set out by Allison [1]. Specifically, for binary variables, the values will be set to zero if the imputed value is less than 0.5 and set to one if the imputed value is greater than or equal to 0.5. For categorical outcomes with *k* levels, imputed values will be obtained for *k – 1* categories, with the final category being omitted as a reference category. The imputed value of the reference category can then be calculated as ${m}_{k}=1- \sum_{i=1}^{k-1}{m}_{i}$, and the imputed categorical variable can be assigned to the category with the largest imputed value, where ${m}_{i}$ is the imputed value for category *i*. Values for imputation of continuous outcomes which fall outside of the plausible range of values will *not* be rounded to within the plausible value range, as such an approach can introduce bias [35, 45]. Appropriate transformations of variables pre-imputation will be considered where necessary to achieve normality, with back-transformation undertaken prior to analysis.

A total of 50 imputed datasets will be generated.

### Mediational analyses

Path analysis models will be fitted to identify whether any of the pre-specified factors outlined in the ‘Pre-specified mediators’ section mediate the effect of the intervention on the primary outcome. Although the primary analysis of the primary outcome is based on a population-averaged model (i.e. using the GEE method), a cluster-specific modelling method, random effects logistic regression, will be used for the purpose of the mediation analyses as the planned Bayesian methodology is not possible using GEEs.

**Fig. 1:**
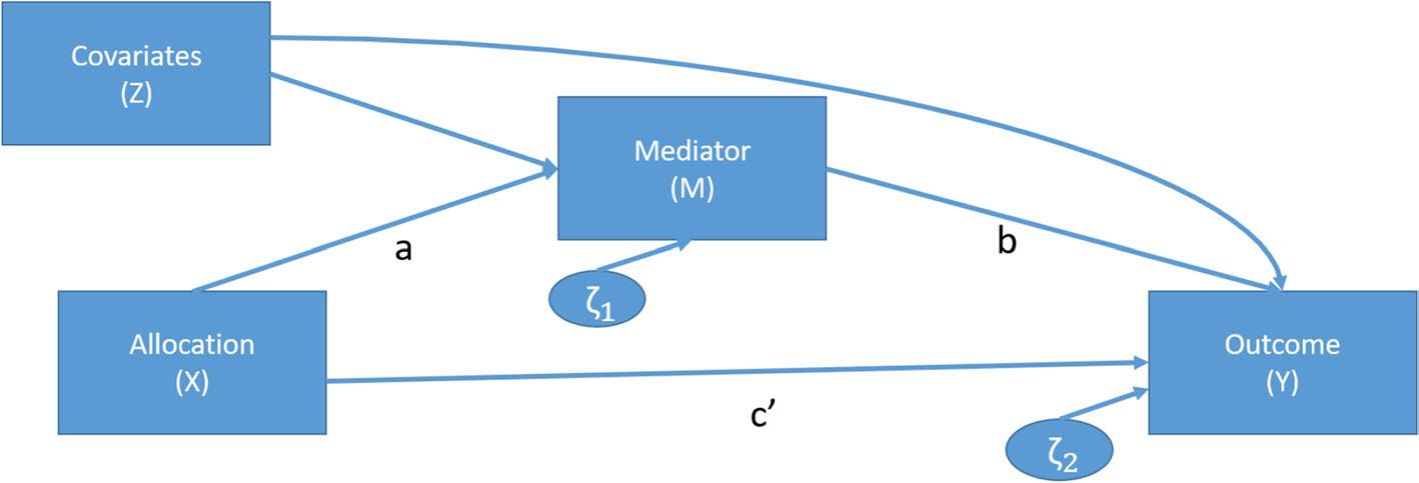
Path diagram of a mediation model

Figure 1 provides a schematic of the planned path analysis modelling strategy, where* X* is the trial arm status, *M* is a mediator, *Y* is the primary outcome (presence of an anxiety disorder at 12 months post-randomisation) and *Z* represents the pre-specified adjustments (school-level free school meal status, cluster size, child gender, child year group, cohort and child socioeconomic status and the measure of the mediator taken at baseline, where available). *a* denotes the effect of the intervention (*X*) on the mediator (*M*) after covariate (*Z*) adjustment, *b* represents the effect of the mediator (*M*) on the outcome (*Y*) (after adjusting for trial arm status (*X*) and the covariates (*Z*)) and *c′* represents the direct effect of the intervention (*X*) on the outcome (*Y*) after adjusting for the effect of the mediator (*M*) and the covariates (*Z*). *c*, not shown in Fig. 1 but included in Eq. 1 below for completeness, denotes the overall effect of the intervention (*X*) on the outcome (*Y*) after adjustment for the covariates (*Z*) (i.e. the ITT estimate). ζ_1_ and ζ_2_ represent the school (cluster)-level random effects on the mediator (*M*) and the outcome (*Y*), respectively. This schematic can be expressed as the following series of equations:

1 $$Y={\delta }_{1}+cX+{R}_{1}Z$$

2 $$M= {\delta }_{2}+aX+{R}_{2}Z$$

3 $$Y= {\delta }_{3}+{c}^{^{\prime}}X+{bM+R}_{3}Z$$

For all planned mediation analyses, *M* is continuous. As a result, Eq. (2) is a linear regression and Eqs. (1) and (3) are logistic regressions (all including random effects at the school (cluster) level). $c$ in Eq. (1) represents the overall effect of the intervention on the outcome; $a$ in Eq. (2) represents the effect of the intervention on the mediator after adjusting for covariates; and ${c}^{^{\prime}}$ and $b$ in Eq. (3) represent the effect of the intervention and the mediator, respectively, on the outcome, after adjusting for covariates. The $\delta$ terms represent the intercept terms for each of the equations, and each of the $R$ terms represents a vector of coefficients associated with the pre-specified covariate adjustments, *Z*.

Following the methods of Krull and MacKinnon [24], the estimate of the indirect (i.e. mediated) effect is calculated as $ab$. The proportion of the overall effect mediated is then calculated as $\frac{ab}{ab+{c}^{^{\prime}}}$.

In order to circumvent the challenges associated with (i) determining an appropriate method of estimating the standard error of the indirect effect in the context of random effects linear and logistic regression models and (ii) appropriately applying Rubin’s rules to synthesise the results of the mediation analysis for the multiply imputed datasets, a Bayesian approach will be adopted. Specifically, Markov Chain Monte Carlo (MCMC) methods will be used to draw samples from the posterior distributions of each of the components of the indirect effect ($a$ and $b$). At each iteration of the MCMC procedure, a sample from the posterior distribution of each of $a$ and $b$ will be obtained. Subsequently multiplying together, these samples of $a$ and $b$ at each iteration will result in samples from the posterior distribution of the indirect effect. A similar approach will be used to obtain posterior samples from the proportion of the overall effect mediated.

Samples from the posterior distributions of each of the indirect effect and the proportion mediated will be obtained for each of the multiply imputed datasets, which can then simply be combined to provide an overall pooled estimate of the indirect effect and proportion mediated [13, 47].

All Bayesian analyses will be fitted using the probabilistic programming language *Stan* [6], which uses Hamiltonian Monte Carlo (HMC) methods [4] to obtain posterior samples. The R package *rstanarm* [16] will likely be used to fit the random effects models, although bespoke *Stan* models may be written if required. Whilst commonly used, non-informative (‘flat’) prior distributions are rarely appropriate as they imply equal or relatively large probability mass to unfeasibly large values of the parameter. As a result, weakly informative (regularising) prior distributions will be specified [11] for all parameters, including the regression coefficients within each model and the variances at both the individual and cluster levels. These will likely be the default prior distributions in the command *stan_lmer* contained within the *rstanarm* package, but the appropriateness of these in the latest package version will be assessed at the point of statistical analysis. All prior distributions will be explicitly reported. Point estimates (means) and 95% credible intervals (CrIs) (calculated using the 2.5% and 97.5% percentiles of the posterior distribution) will be presented for all Bayesian analyses.

In order to aid transparency in the implementation of this non-standard methodology, the code will be made available after the publication of the results.

### Additional analyses

Whilst the primary analysis will be of the imputed data, a sensitivity analysis will be undertaken on the primary and each of the secondary outcomes using complete case data only. An additional sensitivity analysis will be undertaken on the primary outcome to explore the impact of primary outcome data collection occurring outside of the pre-specified window (+12 weeks), including only participants followed up within this window.

For the purposes of a secondary analysis, a CACE analysis of the primary outcome will be undertaken on the basis of the compliance definition outlined in the ‘Adherence and protocol deviations’ section. Specifically, a participant will be classified as a complier if they complete at least the first five online modules (modules 0–4). A CACE analysis facilitates for estimation of the causal effect of *receiving* an intervention on an outcome, in contrast to an ITT analysis which estimates the effect of being *randomised* to the intervention arm. This estimation will be achieved using a two-stage least squares (2SLS) instrumental variable approach, which has been extended from an existing approach [29] to also account for clustering within the data via random effects at stage 1 and via a GEE at stage 2. Specifically, at stage 1, compliance status will be regressed on trial arm status using random effects linear regression, including a school-level random effect, to account for clustering in compliance. At the second stage, a GEE including the same adjustments as specified for the primary analyses, as well as an additional adjustment for the total of the residuals (at the cluster and individual levels), obtained in stage 1 and including a covariate for the compliance status with robust estimates of standard errors (specifying an exchangeable correlation structure) will be used to estimate the CACE. Let ${C}_{i,j}$ denote the compliance status for participant $i$ in cluster $j$, equal to 1 for a complier and 0 for a non-complier. Similarly, let ${Y}_{i,j}$ denote the primary outcome for participant $i$ in cluster $j$, where 1 and 0 represent the presence and absence of an anxiety disorder at 12 months, respectively. Then, mathematically ,the two-stage modelling procedure can be written as:

Stage 1:

$${C}_{i,j}= {\alpha }_{c}+ {\beta }_{c1}{x}_{i,j}+{{\varvec{Z}}}_{1}{{\varvec{\theta}}}_{1}+\boldsymbol{ }{u}_{cj}+ {\varepsilon }_{ci,j}$$

where ${\alpha }_{c}$ is the global intercept term, ${\beta }_{c1}$ is the coefficient for the allocated group, ${x}_{i,j}$ denotes trial arm status (equal to 1 for the intervention arm and 0 for the usual school practice arm),${{\varvec{Z}}}_{1}{{\varvec{\theta}}}_{1}$ denotes the vector of pre-specified adjustments, ${u}_{cj}$ denotes the school (cluster)-level random effects term and ${\varepsilon }_{ci,j}$ denotes the individual-level error term.

Stage 2:

$$\mathrm{log}\left(\frac{{p}_{i,j}}{1- {p}_{i,j}}\right)= \alpha + {\beta }_{1}{C}_{i,j}+{\beta }_{2}\widehat{{\varepsilon }_{Ci,j}}+ {{\varvec{Z}}}_{2}{{\varvec{\theta}}}_{2}$$

$${Y}_{i,j}\sim Binomial(1,{p}_{i,j})$$

where $\alpha$ is the intercept term, ${p}_{i,j}$ is the probability of the presence of an anxiety disorder at 12 months for participant $i$ in cluster $j,$ ${C}_{i,j}$ is the observed compliance status, $\widehat{{\varepsilon }_{Ci,j}}$ are the total of the cluster- and individual-level residuals from the stage 1 model, ${{\varvec{Z}}}_{2}{{\varvec{\theta}}}_{2}$ denotes the vector of pre-specified adjustments and the CACE estimator of interest is ${\beta }_{1}$.

Standard errors for the CACE estimate (${\beta }_{1}$) will be obtained using cluster-level bootstrapping of the 2SLS procedure using the *cluster* option within Stata’s *bootstrap* command. Additional predictors of compliance may be considered for inclusion in stage 1 of the 2SLS procedure. Rubin’s rules will be used to estimate the standard error of the CACE estimator across the imputed datasets.

In order to aid transparency in the implementation of this non-standard methodology, the code will be made available online after the publication of the results.

### Harms

Any potential adverse events will be recorded and managed in accordance with the study adverse event protocol. Causality will be assessed, and adverse events directly related to participation in the study will be categorised as serious (serious adverse event) or not serious (adverse event).

### Statistical software

The majority of the statistical analysis will be undertaken using Stata v17.0 or higher [42]. The multiple imputation will be undertaken using R v3.6.1 [33] or higher using the packages *pan* and *mitml* [17] and analysed using the *mi* suite of commands in Stata. The randomisation lists were generated in R.

## Discussion

This statistical analysis plan prospectively describes the planned analyses for the MY-CATS cluster randomised controlled trial and was written and approved by the trial statistician, the independent statistician and the chief investigator during the delivery phase of the trial and therefore prior to database lock. Given the planned analyses include non-standard methodology, including Bayesian path analysis models and instrumental variable methods using cluster-level bootstrapping, the SAP has been released as a standalone publication in line with recent recommendations [18].

## Data Availability

Datasets and study materials generated during the current study will be made available in a public repository.

## Abbreviations

ADIS-P: Anxiety Disorder Interview Schedule—Child Version—Parent Interview
AT: As treated
BI: Behavioural inhibition
CACE: Complier Average Causal Effect
CALIS-PV: Child Anxiety Life Interference Scale—Preschool version
CAMP: Child Avoidance Measure—Parent Report
CHU-9D: Child Health Utility—9 Dimension
CI: Confidence interval
CQ-P: Child Coping Questionnaire—Parent Report
CSRI: Client Service Receipt Inventory
EQ-5D-5L: EuroQol 5 Dimension 5 Level instrument
GAD: Generalised anxiety disorder
GEE: Generalised estimating equation
HMC: Hamiltonian Monte Carlo
ICC: Intra-cluster correlation coefficient
ITT: Intention-to-treat
MCMC: Markov Chain Monte Carlo
OSI: Online Support and Intervention for child anxiety
RULES: Responses to Uncertainty and Low Environmental Structure
PAS: Preschool Anxiety Scale
POS: Parental Overprotection Scale
PP: Per protocol
PSCS: Parent Sense of Competence Scale
SDQ: Strengths and Difficulties Questionnaire
STSC: Short Temperament Scale for Children
